# Clinical Accuracy, Relevance, Clarity, and Emotional Sensitivity of Large Language Models to Surgical Patient Questions: Cross-Sectional Study

**DOI:** 10.2196/56165

**Published:** 2024-06-07

**Authors:** Mert Marcel Dagli, Felix Conrad Oettl, Jaskeerat Gujral, Kashish Malhotra, Yohannes Ghenbot, Jang W Yoon, Ali K Ozturk, William C Welch

**Affiliations:** 1 Department of Neurosurgery University of Pennsylvania Perelman School of Medicine Philadelphia, PA United States; 2 Department of Orthopedic Surgery Hospital for Special Surgery New York, NY United States; 3 Department of Orthopedic Surgery Schulthess Clinic Zurich Switzerland; 4 Institute of Applied Health Research University of Birmingham Birmingham United Kingdom

**Keywords:** artificial intelligence, AI, natural language processing, NLP, large language model, LLM, generative AI, cross-sectional study, health information, patient education, clinical accuracy, emotional sensitivity, surgical patient, surgery, surgical

## Abstract

This cross-sectional study evaluates the clinical accuracy, relevance, clarity, and emotional sensitivity of responses to inquiries from patients undergoing surgery provided by large language models (LLMs), highlighting their potential as adjunct tools in patient communication and education. Our findings demonstrated high performance of LLMs across accuracy, relevance, clarity, and emotional sensitivity, with Anthropic’s Claude 2 outperforming OpenAI’s ChatGPT and Google’s Bard, suggesting LLMs’ potential to serve as complementary tools for enhanced information delivery and patient-surgeon interaction.

## Introduction

Recent advances in natural language processing (NLP) have produced large language model (LLM) applications, such as OpenAI’s ChatGPT, that have captivated a worldwide audience [1]. They have permeated the health care sector, offering several benefits [2]. While LLMs have immense potential in improving clinical practice and patient outcomes, their role has not been completely established [3]. Often, patients that require surgery struggle with complex, anxiety-inducing questions [4]. Thus, counseling during preoperative workup is crucial for obtaining informed consent, establishing trust, and ensuring presurgical optimization to improve patient outcomes. This process, being resource-intensive and involving numerous conversations, often delays communication, causing significant frustration for patients [5]. Therefore, the importance of clear, adequate, and timely information delivery cannot be overemphasized. LLMs with chat features could improve preoperative communication; however, LLMs’ ability in answering patients’ surgical questions have not been extensively studied. Thus, this study aims to assess LLMs’ potential and proficiency in responding to questions from patients undergoing surgery.

## Methods

### Overview

In formulating our questionnaire, we used the input of 3 neurosurgical attendings, focusing on common general patient inquiries regarding surgery. We presented 38 patient questions in web sessions to 3 publicly accessible LLMs: ChatGPT (GPT-4; OpenAI), Claude 2 (Anthropic), and Bard (Google) on August 16, 2023 (Multimedia Appendix 1). Questions had 4 central themes: the nature and rationale of a surgery, preoperative concerns, procedural aspects, and postoperative considerations. Each reply from the LLMs was reviewed by 2 independent blinded reviewers (MMD and FCO, research fellows with medical doctorates who had not completed postgraduate clinical training). A 5-point Likert scale was used to assess accuracy, relevance, and clarity of responses [6]. Emotional sensitivity was evaluated on a 7-point Likert scale to increase discriminatory power [7]. Assessment of data normality used the Shapiro-Wilk test. Homogeneity of variances (homoscedasticity) across groups was evaluated via the Levene test. For nonparametric analysis, the Kruskal-Wallis test was used to discern differences among groups. Subsequent pairwise comparisons were facilitated by the post hoc Dunn test. In instances where parametric assumptions were upheld, a 1-way ANOVA was conducted, followed by post hoc analysis with the Tukey honestly significant difference (HSD) test. *P* values from the post hoc analysis were adjusted for multiplicity with Bonferroni correction. Additionally, weighted percentage agreement (WPA) was used to determine agreement between raters. All statistical analyses used Python (version 3.7; Python Foundation).

### Ethical Considerations

The study qualified for institutional review board exemption as it exclusively used questions sourced from surgeon input, with no direct patient involvement.

## Results

Shapiro-Wilk testing indicated nonnormality (*P*<.05; Table 1) for accuracy, relevance, and clarity scores. Levene testing revealed nonhomoscedasticity for relevance (*F*_2_=5.009; *P*=.01). The Kruskal-Wallis test showed significant differences in the distribution of accuracy (*H*=27.464; *P*<.001), relevance (*H*=29.074; *P*<.001), and clarity (*H*=32.745; *P*<.001). The post hoc Dunn test demonstrated that Claude 2’s responses were significantly more highly rated than ChatGPT’s or Bard’s for accuracy, relevance, and clarity (*P*<.05). There were no significant differences between ChatGPT and Bard except in clarity (*Z*=1.972; *P*=.04). ANOVA showed significant differences in emotional sensitivity (*F*_2,111_=10.799; *P*<.001). The post hoc Tukey HSD test revealed significantly higher emotional sensitivity scores for Claude 2 compared to ChatGPT and Bard (*P*<.05). WPA was highest for Claude 2, followed by ChatGPT and Bard (Tables 2 and 3).

**Table 1:** Results of normality test (Shapiro-Wilk), homoscedasticity test (Levene), nonparametric test (Kruskal-Wallis), post hoc pairwise comparison of nonparametric data (Dunn test with Bonferroni correction), parametric test (ANOVA), and post hoc pairwise comparison of parametric data (Tukey honestly significant differences [HSD] test with Bonferroni correction).

Test			Value	*P* value
**Shapiro-Wilk (*W* statistic)**				
	**Accuracy**			
		ChatGPT	0.862	<.001
		Claude 2	0.711	<.001
		Bard	0.87	<.001
	**Relevance**			
		ChatGPT	0.845	<.001
		Claude 2	0.604	<.001
		Bard	0.917	.01
	**Clarity**			
		ChatGPT	0.886	.01
		Claude 2	0.747	<.001
		Bard	0.933	.02
	**Emotional sensitivity**			
		ChatGPT	0.965	.27
		Claude 2	0.953	.11
		Bard	0.959	.18
**Levene (*F*_2_ statistic)**				
	Accuracy		2.144	.12
	Relevance		5.009	.01
	Clarity		1.918	.15
	Emotional sensitivity		0.184	.83
**Kruskal-Wallis (*H* statistic)**				
	Accuracy		27.363	<.001
	Relevance		29.074	<.001
	Clarity		32.745	<.001
**Dunn test with Bonferroni (*Z* statistic)**				
	**Accuracy**			
		ChatGPT vs Claude 2	–2.546	.01
		ChatGPT vs Bard	1.56	.15
		Claude 2 vs Bard	4.106	<.001
	**Relevance**			
		ChatGPT vs Claude 2	–2.872	<.001
		ChatGPT vs Bard	1.235	.34
		Claude 2 vs Bard	4.107	<.001
	**Clarity**			
		ChatGPT vs Claude 2	–2.546	.01
		ChatGPT vs Bard	1.972	.04
		Claude 2 vs Bard	4.518	<.001
*F* statistic (*df*) from ANOVA (for emotional sensitivity)			10.799 (2,111)	<.001
**Tukey HSD test with Bonferroni (emotional sensitivity; *Q* statistic)**				
	ChatGPT vs Claude 2		–0.974	<.001
	Bard vs ChatGPT		0.21	.60
	Claude 2 vs Bard		0.763	.01

**Table 2:** Adjusted percentage average ratings of large language model responses. Adjusted average percentage ratings were calculated as the mean of normalized scores using the following formula to scale responses uniformly from 0% to 100%: adjusted percentage rating = ((actual Likert score – 1) / (Likert scale maximum – 1)) × 100%.

	ChatGPT			Claude 2			Bard	
	Likert score, mean (SD)	Adjusted average Likert rating (%), mean (SD)	Likert score, mean (SD)		Adjusted average Likert rating, mean (SD)	Likert score, mean (SD)		Adjusted average Likert rating, mean (SD)
Accuracy	4.2 (0.55)	79.93 (13.8)	4.61 (0.58)		90.13 (14.58)	3.76 (0.85)		69.08 (21.3)
Relevance	4.28 (0.64)	81.91 (16.1)	4.76 (0.4)		94.08 (9.96)	4.04 (0.67)		75.99 (16.79)
Clarity	4.24 (0.61)	80.92 (16.1)	4.68 (0.38)		92.11 (9.38)	3.86 (0.64)		71.38 (15.89)
Emotional sensitivity	4.49 (1)	58.11 (16.61)	5.46 (0.92)		74.34 (15.3)	4.7 (0.97)		61.62 (16.16)

**Table 3:** Weighted percentage agreement (WPA) point estimates.

	ChatGPT, WPA (95% CI)	Claude 2, WPA (95% CI)	Bard, WPA (95% CI)
Accuracy	80.26 (67.61-92.92)	86.84 (76.09-97.59)	71.05 (56.63-85.47)
Relevance	76.32 (62.8-89.83)	97.37 (92.28-102.46)	71.05 (56.63-85.47)
Clarity	72.37 (58.15-86.59)	94.74 (87.64-101.84)	60.53 (44.98-76.07)
Emotional	68.42 (53.64-83.2)	77.63 (64.38-90.88)	67.11 (52.17-82.04)

## Discussion

### Principal Findings

Our investigation revealed potential for using LLMs in patient education. Claude 2 had significantly higher percentage average ratings of above 90% for accuracy (*P*=.004 and *P*<.001), relevance (*P*<.001), and clarity (*P*=.004 and *P*<.001) compared to ChatGPT and Bard. It also scored significantly better on emotional sensitivity than ChatGPT and Bard (*P*<.001 and *P*=.01), with 74.3%. In a study parallel to ours, Sezgin et al [8] assessed the clinical accuracy of LLMs in the context of postpartum depression, demonstrating their efficacy in providing clinically accurate information, a finding that complements our study’s illustration of LLMs’ potential in patient education and engagement. By providing accurate and timely information, LLMs can potentially alleviate patient concerns.

### Limitations

The study’s limitations include the absence of direct patient input when formulating the questionnaire, the lack of repeated zero-shot questioning, which may reveal variability, and no dedicated analysis of overtly inaccurate “hallucinations.” The principal challenge for LLM deployment in clinical settings lies in its regulatory approval and secure integration within health care systems [9]. We are actively conceptualizing a randomized clinical trial controlling for these limitations to investigate LLM and surgeon responses as rated by patients and surgeons.

### Conclusions

While surgeons remain indispensable in patient education, LLMs can potentially serve as a complementary tool, enhancing information delivery and supporting patient-surgeon interactions.

## Appendix

Multimedia Appendix 1 Responses to surgical patient questions.

## Abbreviations

HSD: honestly significant difference
LLM: large language model
NLP: natural language processing
WPA: weighted percentage agreement

## Data Availability

All data generated or analyzed during this study are included in this published article and its multimedia appendix.
